# The choroid plexus: a door between the blood and the brain for tissue-type plasminogen activator

**DOI:** 10.1186/s12987-022-00378-0

**Published:** 2022-10-15

**Authors:** Vincent Zuba, Jonathane Furon, Mathys Bellemain-Sagnard, Sara Martinez de Lazarrondo, Laurent Lebouvier, Marina Rubio, Yannick Hommet, Maxime Gauberti, Denis Vivien, Carine Ali

**Affiliations:** 1 Physiopathology and Imaging of Neurological Disorders, Normandie Univ, UNICAEN, INSERM, INSERM UMR-S U1237, Institut Blood and Brain @ Caen-Normandie, GIP Cyceron, Boulevard Becquerel, 14074 Caen, France; 2 Department of Clinical Research, Caen-Normandie Hospital (CHU), Caen, France

**Keywords:** Tissue-type plasminogen activator, Choroid plexus, Epithelium, Low density lipoprotein receptor-related protein, Barrier, Transport, Finger domain, Drug delivery

## Abstract

**Background:**

In the vascular compartment, the serine protease tissue-type plasminogen activator (tPA) promotes fibrinolysis, justifying its clinical use against vasculo-occlusive diseases. Accumulating evidence shows that circulating tPA (endogenous or exogenous) also controls brain physiopathological processes, like cerebrovascular reactivity, blood–brain barrier (BBB) homeostasis, inflammation and neuronal fate. Whether this occurs by direct actions on parenchymal cells and/or indirectly via barriers between the blood and the central nervous system (CNS) remains unclear. Here, we postulated that vascular tPA can reach the brain parenchyma via the blood-cerebrospinal fluid barrier (BCSFB), that relies on choroid plexus (CP) epithelial cells (CPECs).

**Methods:**

We produced various reporter fusion proteins to track tPA in primary cultures of CPECs, in CP explants and in vivo in mice. We also investigated the mechanisms underlying tPA transport across the BCSFB, with pharmacological and molecular approaches.

**Results:**

We first demonstrated that tPA can be internalized by CPECs in primary cultures and in ex vivo CPs explants. In vivo, tPA can also be internalized by CPECs both at their basal and apical sides. After intra-vascular administration, tPA can reach the cerebral spinal fluid (CSF) and the brain parenchyma. Further investigation allowed discovering that the transcytosis of tPA is mediated by Low-density-Lipoprotein Related Protein-1 (LRP1) expressed at the surface of CPECs and depends on the finger domain of tPA. Interestingly, albumin, which has a size comparable to that of tPA, does not normally cross the CPs, but switches to a transportable form when grafted to the finger domain of tPA.

**Conclusions:**

These findings provide new insights on how vascular tPA can reach the brain parenchyma, and open therapeutic avenues for CNS disorders.

**Supplementary Information:**

The online version contains supplementary material available at 10.1186/s12987-022-00378-0.

## Background

The blood–brain barrier (BBB) has often been considered as being “the” barrier of the CNS. However, over the past 10 years, choroid plexuses (CP) have (re)emerged as another critical interface, fulfilling essential functions for CNS homeostasis [43]. Floating in brain ventricles, CPs were originally described as being responsible for the secretion of the cerebrospinal fluid (CSF), with critical consequences on intracranial pressure [12]. Among others, under physiological conditions, CPs also have functions of sensor, neurotrophic niche, and regulator of some behaviours [2, 17, 34, 36]. CPs participate in the pathogenesis and progression of various neurological diseases as well as in the spread of inflammatory responses from the periphery to the CNS [5, 9, 20, 22, 28, 31, 33, 35, 44, 46].

CPs consist in a densely vascularized matrix, the stroma, populated by immune cells and surrounded by epithelial cells. Thanks to their apical tight junctions, the choroid plexus epithelial cells (CPECs) form a physical barrier at the interface between the blood and the CSF [13]. Owing to the numerous transporters expressed by CPECs, the blood-CSF barrier (BCSFB) is a real hub of exchanges [38]. The BCSFB also stands as a target for drug delivery and/or for prevention of entry/accumulation of toxic mediators [6, 37].

In this study, we were interested in a key fibrinolytic protease, tissue-type plasminogen activator (tPA). When released in the bloodstream from endothelial cells and/or hepatocytes [26, 48], tPA can cleave plasminogen into active plasmin, leading to the degradation of fibrin clots [10]. Accordingly, the recombinant form of tPA (Actilyse, Alteplase) is of therapeutic value for several vasculo-occlusive conditions, including pulmonary embolism, deep vein thrombosis and ischemic stroke [18, 21, 45]. Beside fibrinolysis, circulating tPA (endogenous and exogenous) exerts various effects in/on the normal or pathological CNS (for review, see [42]. Some of these cerebral effects of circulating tPA occur by direct actions on the vessel wall. For instance, physiological neurovascular coupling, a phenomenon that links neuronal activity to cerebral blood flow, is tuned by intraluminal tPA acting on N-methyl-D-aspartate receptors (NMDAR) on endothelial cells [1]. Conversely, under pathological conditions, this tPA/NMDAR endothelial partnership promotes BBB leakage and inflammation [29]. Beside these direct effects on the brain vasculature, tPA can also “escape” from the blood [3, 4], and add to parenchymal tPA to increase neuronal activity and neuronal loss [32]. This escape was thought to result from the polarized trans-endothelial transport of tPA, driven by Low-density-Lipoprotein Related Protein-1 (LRP1), a member of the LDL receptor-related protein family [3, 4].

In light of the recent literature about the BCSFB, it is relevant to determine whether—and if so, how—vascular tPA also crosses the CPs. In the present study, we showed that CPECs can uptake tPA coming from the blood or from the CSF and that, CPECs transport vascular tPA and translocate it into the CSF. With in vitro and in vivo models, we showed that the influx of tPA is an active mechanism relying on LRP1 at the surface of CPECs. Interestingly, we identified the finger domain of tPA as being involved in this uptake, and that grafting this finger domain to albumin, allows its passage through CPECs. Our findings open interesting avenues for new strategies of drug delivery and maybe for a safer use of tPA during stroke, as blocking its entry in the brain parenchyma would prevent its pro-neurotoxic effect without affecting its beneficial thrombolytic action.

## Materials and methods

### ***Labelling of recombinant proteins with AlexaFluor***^***®***^

Apart from BSA coupled to AlexaFluor^488^ or AlexaFluor^555^, which were commercially available (Life Technologies), recombinant proteins were conjugated to AlexaFluor^488^ or AlexaFluor^555^ as follows. For tPA, Actilyse^®^ (Boehringer Ingelheim) was first dialyzed in a 0.3 M bicarbonate buffer, pH 8.4, at 4° C for 48 h, in order to remove excipients, in particular arginine (the dialysis buffer was changed every twelve hours). Next, recombinant proteins were mixed with 1 mg of AlexaFluor^555^ (λ_excitation_: 555 nm; λ_emission_: 592 nm) or AlexaFluor^488^ (λ_excitation_: 494 nm and λ_emission_: 517 nm) Succinimidyl Ester (Life Technologies), diluted in 100μL of dimethyl sulfoxide, for 6 h at room temperature and with constant stirring. In order to remove excess AlexaFluor, the solution was dialyzed in a bicarbonate buffer first, and then in a phosphate buffered saline containing arginine hydrochloride to avoid protein precipitation.

### Production of recombinant ΔF-tPA and F-BSA

HEK293/FRT cells were used as the eukaryotic system for production of recombinant proteins with the Flp-in system (ThermoFisher scientific). After selection with hygromycin B, stable cells were placed in production without serum (60 petri dishes per protein, diameter 10 cm), culture media were changed every 4 days for 12 days. The PCDNA3.1-Rattus tPA delta finger construct was made from the PCDNA3.1-Rattus-tPA vector. The latter is constructed like this: ATG-6His-BamhI-tPA-Stop-XhoI. tPA delta finger was amplified by PCR from amino acid 79 of tPA of PCDNA3.1-Rattus tPA (beginning of the EGF like domain) to the stop and cloned between BamHI and XhoI of the PCDNA3.1-Rattus tPA construct. We thus obtained ATG-6His-BamhI-tPA delta finger-Stop-XhoI. The human albumin cDNA was purchased from GenScript (vector pcDNA3.1 + /C-(K)DYK-Albumin, #OHu18744). We amplified the cDNA of the finger domain by PCR from a PCDNA5.1-Rattus tPA construct (finger: 36- > 78), the domain was amplified with ATG, the signal peptide for secretion and the 6his tag of the PCDNA5.1-Rattus tPA construct. It was then cloned between NheI and HindIII of the pcDNA3.1 + /C-(K)DYK-Albumin vector. Finally we obtained an amino terminal cloning: ATG-6His-Finger-Albumin-Stop.

The 6xHistidine fused to the N-terminal of ΔF-tPA and of F-BSA allows purification of recombinant proteins by high performance liquid chromatography using Nickel-Nitrilo-triAcetic Acid affinity columns (Ni–NTA, GE Healthcare) and a mobile phase imidazole gradient. Positive elutions were pooled, concentrated and dialyzed in phosphate buffer. The ΔF-tPA and F-BSA proteins were then coupled to AlexaFluor^488^ and AlexaFluor^555^, respectively, as described above, except that there was no initial dialysis.

### Animals

Eight weeks old male Swiss mice (35–45 g, Centre Universitaire de Ressources Biologiques, Normandy University, Caen, France) were housed at 21 °C in a 12-h light/dark cycle with food and water ad libitum. All procedures with anaesthesia were performed by an initial exposure to 5% isoflurane, followed by a maintaining phase of 1.5–2% isoflurane 30%O_2_/70%N_2_O.

### Intravenous (IV) Injection of Alexa-coupled recombinant proteins, CSF sampling and tissue collection

Anesthetized mice were placed in a stereotaxic frame. Recombinant proteins were injected in the tail vein, in 200µL of saline (0.9% NaCl) over approximately 5 s.

Following the IV injection, the mice were kept anesthetized in the stereotaxic frame and monitored while awaiting for the sampling of CSF and brain. Lidocaine (a local pain reliever) was applied on the incision plan 5 min before starting surgery. The upper muscular planes of the neck were detached and spread apart in order to unmask the *cisterna magna*. Once the cistern was unmasked, a glass micropipette was inserted in order to collect the CSF (up to 7-8μL), which was transferred in a tube on dry ice and stored at − 80 °C. Mice were immediately subjected to intracardiac perfusion of heparinized saline at 4 °C followed by a phosphate buffer solution, 0.1 M and pH 7.4, with 4% paraformaldehyde. Following perfusion, the brain and cerebellum were extracted and placed in a phosphate buffered saline (PBS) solution containing 20% sucrose for 48 h (renewed four times). Tissues were included in Cryomatrix™ (Thermo Scientific) and stored at − 80 °C. Ten μm thick sections were made using a cryostat (Leica, CM3050S), placed on poly-lysine slides and were stored at − 80 °C.

### Immunohistochemistry

Tissue sections were rinsed and rehydrated in PBS and are then incubated in a PBS solution containing 0.25% of Triton X-100 overnight at room temperature and in a humid atmosphere, with an anti-TTR antibody to label the CPECs (1:1000; Abcam 9015) or anti.

CD-31, a marker of the endothelium (1:1000; BD Pharmingen 553,370). The next day sections were incubated in PBS containing 0.25% Triton ™ X-100 and Fab'2 fragments directed against the host species primary antibody (1:1000; Jackson ImmunoResearch) for 90 min at room temperature and humid atmosphere. Sections were covered with an assembly medium (Fluoromount-G^®^, SouthernBiotech) containing, or not, DAPI and a coverslip. The slides were then observed and microphotographed with an inverted confocal microscope (Leica TCS SP8) to be analysed with Fiji Is Just ImageJ. For each animal, a dozen images were acquired on 5 Sects. (4 of the brain and one of the cerebellum). On each image, the CPECs were manually counted, thanks to TTR immunostaining, as well as the spots of fluorescence within CPECs.

A subset of slices was also stained with an antibody against NeuN (mouse monoclonal antibody MAB377; 1:500; Merck Millipore, Alexa^647^-conjugated secondary antibody donkey anti-mouse, 1:800, Jackson ImmunoResearch) to determine if tPA^555^ can be detected in the brain parenchyma (30 min after intravenous administration).

### tPA/BSA levels in the CSF

After addition of loading buffer (Sucrose, SDS, Bromophenol Blue), the CSF samples (5 µl) were subjected to electrophoresis (SDS-PAGE) under non-reducing conditions (200 V, 50 min) on a 10% polyacrylamide gel. The fluorescence signal was measured in the gel using an ImageQuant™ LAS 4000 camera (GE Healthcare), and the signal quantification was done with Fiji is just ImageJ. Alternatively, tPA was immunodetected after transfer onto a PVDF membrane, using a goat anti-tPA antibody (1:500) and a peroxydase-conjugated secondary antibody (1/160000).

### Primary cultures of CPECs

The CPECs were isolated based on a protocol described in rats by Monnot & Zheng [30], with some modifications. After cervical dislocation, preceded by deep anaesthesia with 5% isoflurane (in 30% O_2_ / 70% N_2_O), and recovery of the brains of ten 8-week-old Swiss mice, the plexuses from 3rd and 4th ventricles and lateral ventricles were dissected under a binocular lens. The plexuses were diluted in 1 ml of ice-cold HBSS (Hank's Balanced Salt Solution, Sigma) and underwent enzymatic digestion with pronase (Pronase E from Streptomyces griseus, Serva Electrophoresis) at 2 mg/ml for 20 min at 37 °C and 5% CO_2_. Digestion was stopped by adding 4 mL of ice-cold HBSS. The plexus clusters were centrifuged at 4 °C for 5 min at 800*g*, the supernatant, which mainly contains debris, was removed, the pellet suspended in 6 ml of ice-cold HBSS, centrifuged again at 800 g for 5 min. The pellet was then suspended in 2 ml of growth medium (sodium pyruvate 110 μg/ml, glutamine 2 mM, fetal calf serum 10%, epidermal growth factor 0.01 mg/ml and 1% of diluted penicillin/streptomycin in Essential Medium Modified by Dulbecco, DMEM) at 37 °C then mechanically dissociated using a syringe with a 21G needle. The dissociated cells were firstly seeded in a 35 mm dish and incubated at 37 °C and 5% CO_2_ for 4 h so that the fibroblasts and macrophages adhere to the plastic. After 4 h, CPECs in suspension in the dish were collected and seeded in 24-well plates previously treated with type I collagen from rat tail (Cultrex ™) at 0.1 mg/mL in acetic acid 0.02 M. After 48 h, the plates were rinsed with PBS and the medium was replaced by medium supplemented with cytosine arabinoside (Ara-C). Cells were then rinsed, and the medium changed every other day until use after 8 days in vitro. The continued presence of the inhibitor of DNA synthesis Ara-C specifically inhibits the growth of contaminating cells such as stromal fibroblasts. Unlike CPECs, these cells express nucleoside transporters unable to distinguish ribose and arabinose residues and thus incorporate Ara-C into their genomic DNA, which results in specific suppression of their growth [25].

### Internalization of tPA by cultured CPECs

Cultured CPECs were rinsed twice with PBS at 37 °C, then maintained for 1 h in a serum-free medium, and treated with tPA^555^ (at the indicated doses/durations). After treatment, cells were rinsed three times with cold PBS. Proteins were extracted at 4 °C in a 50 mM Tris–HCl buffer, pH 7.4, 150 mM NaCl and 0.5% Triton ™ X-100 (TNT buffer) supplemented with protease and phosphatase inhibitors 1 µg/mL. After centrifugation (12000*g* at 4 °C for 20 min), the supernatants were collected and then assayed using the Pierce BCA Protein assay kit. After adding loading buffer (Sucrose, SDS, Bromophenol Blue), the total proteins were separated according to their molecular mass by electrophoresis (SDS-PAGE) on a 10% polyacrylamide gel under non-reducing conditions (200 V, 50 min). The fluorescence of tPA^555^ was detected directly in the gel with an ImageQuant™ LAS 4000 camera, and the signal quantification was done with Fiji Is Just ImageJ.

### RNA silencing

LRP1 Stealth RNAi™ oligoribonucleotides were transfected at a concentration of 20 nM into CPECs cultures using Lipofectamine 2000 (2 µg, Invitrogen). Sequences were: siLRP1_01 sense UGGCUGACGGGAAACUUCUACUUUG; siLRP1_01 antisense CAAAGUAGAAGUUUCCCGUCAGCCA; siLRP1_02 sense CACACCCAUUUGCCGUGACACUGU; siLRP1_02 antisense UACAGUGUCACGGCAAAUGGGUGUG; siLRP1_03 sense CCAAGGUGUGAGGUGAACAAGUGUA; siLRP1_03 antisense UACACUUGUUCACCUCACACCUUGG.

Stealth RNAi™ siRNA Negative Control, Medium GC negative control duplex (Invitrogen™) was used as the control condition (scramble). After 48 h, cells were rinsed 3 times with PBS, and total proteins were recovered. The efficacy of extinction was checked by western blot: after adding loading buffer (Sucrose, SDS, Bromophenol Blue), the total proteins were separated according to their molecular mass by electrophoresis (SDS-PAGE) on a 10% polyacrylamide gel under non-reducing conditions (200 V, 50 min). Then, Proteins were transferred (120 min, 200 mA) onto a cellulose membrane in a humid environment (24 mM Tris, 192 mM glycine, 20% ethanol). Membranes were incubated in a 2% TTBS-BSA solution (Tween Tris Buffered Saline, 10 mM Tris, 200 mM NaCl, 0.1% Tween—2% BSA, pH 7.4) for 1 h and then incubated overnight at 4 °C with the primary anti-LRP1 antibody (Abcam 92,544) diluted 1:1000 in the TTBS-BSA 2% buffer. Membranes were incubated with the secondary antibody coupled to peroxidase, diluted 1:50,000 in 2% TTBS-BSA, for 1 h at room temperature. The detection was performed using the Pierce ECL Plus Western blotting substrate kit, membranes were then placed in the ImageQuant ™ LAS 4000 camera and the signal quantification done with Fiji Is Just ImageJ.

### Internalization of recombinant proteins on CPs explants

Whole CPs were collected from mice (8-weeks-old Swiss males) and were quickly washed with PBS at 37 °C. They were then exposed for the indicated time to the specified Alexa-conjugated protein. After treatment PCs were rinsed three times with PBS and fixed with 4% paraformaldehyde in PBS 0.1 M and pH 7.4, for 15 min. PCs were mounted in Fluoromount-G® then micro-photographed with an inverted confocal microscope (Leica TCS SP8) to be then analysed with Fiji Is Just ImageJ. The various proteins were quantified by their fluorescence within the CPECs, which were delimited manually.

### Statistical analyses

Results are expressed as mean ± the Standard Error of the Mean (SEM). The samples being unpaired and having low numbers a Mann–Whitney U test was used. Statistical analyses were performed using Mann–Whitney test with GraphPad software. Data were considered statistically different if probability values (p) were at least < 0.05.

## Results

### Choroid plexus epithelial cells transport tPA: in vitro and in vivo demonstrations of influx and efflux pathways

**Fig. 1:**
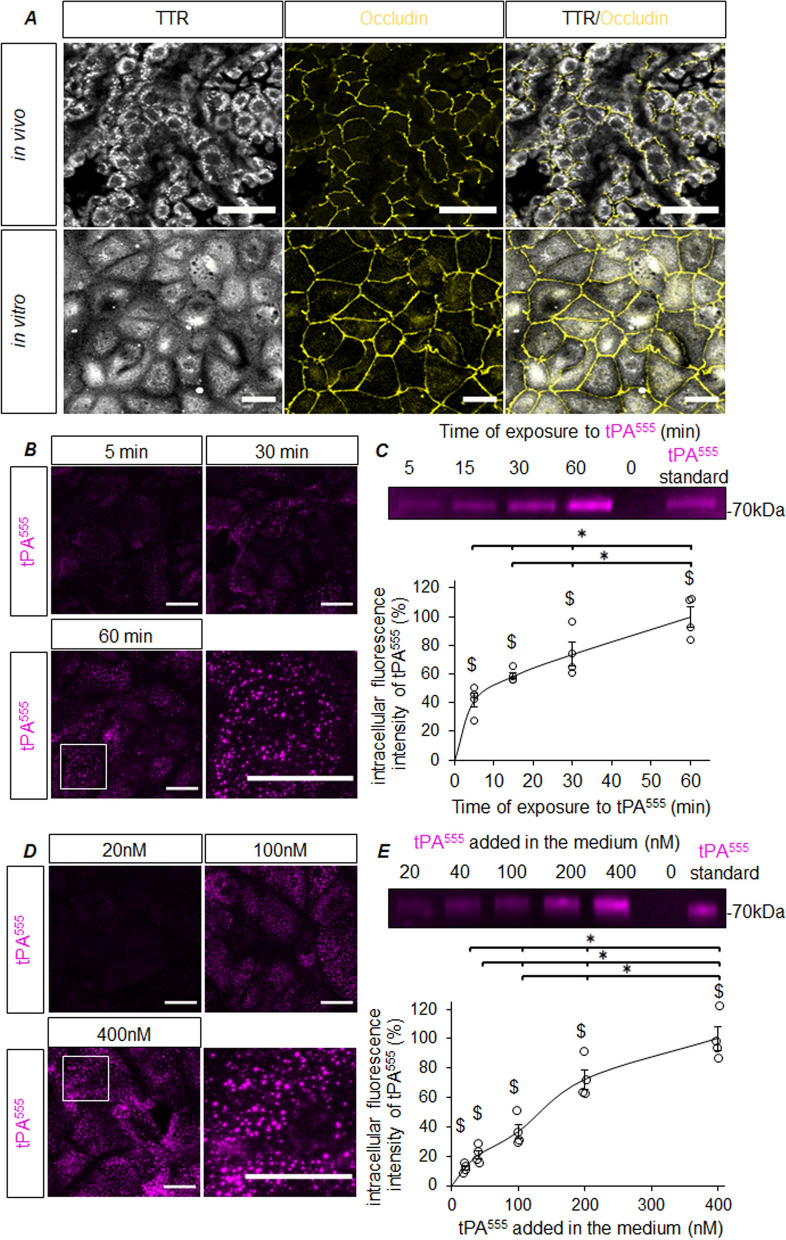
CPECs uptake tPA in vitro.** A** Photomicrographs show that both CPECs in culture (top panel) and CPECs in situ (lower panel) express the marker TTR (white) and tight junctions (Occludin, yellow), scale bar 30 µm. **B**–**E** Primary cultures of CPECs were exposed to 40 nM tPA^555^ for 5-60 min (**B**–**C**) or to tPA^555^ (20-400 nM) for 15 min (**D**–**E**). **B**, **D** Photomicrographs (representative of n = 4) show time- and dose-dependent accumulation of tPA^555^ (magenta) in cell monolayers. Higher magnifications suggest an accumulation in vesicles; scale bar 40 µm. **C**, **E** Electrophoresis (representative of n = 4) and corresponding quantifications of fluorescence (expressed as percentage of maximal uptake; mean ± SEM; n = 4) of protein extracts from monolayer of CPECs exposed to tPA^555^ (40 nM) for 5-60 min (**B**–**C**) or to 20-400 nM tPA^555^ for 15 min (**D**–**E**). * Significant difference (*p* < 0.05), $ significant difference versus control (*p* < 0.05)

**Fig. 2:**
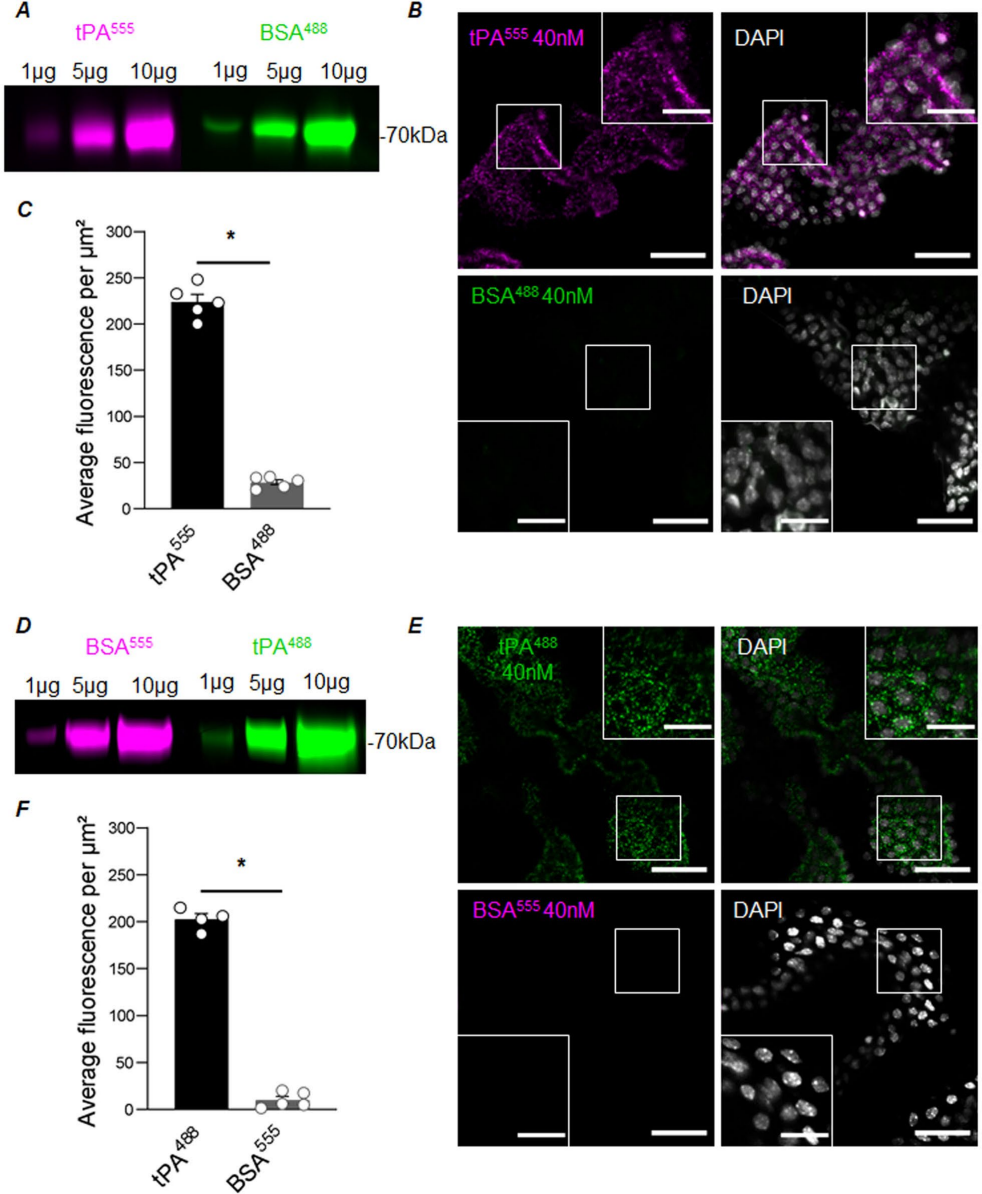
CPECs uptake tPA ex vivo. Freshly isolated CP explants were treated for 15 min with tPA and BSA (40 nM each), alternatively coupled to Alexa^555^ or Alexa^488^. **A** Dose-dependent fluorescent signal generated by tPA^555^ (magenta) and BSA^488^ (green) after SDS-PAGE. **B** Representative confocal photomicrographs of CP explants exposed 15 min to 40 nM of tPA^555^ (magenta), BSA^488^ (green) and DAPI staining (white). Scale bar 40 µm and 30 µm in the magnified inserts. **C** Graph shows quantification of fluorescence in CP explants (n = 5, mean ± SEM), * Significant difference (*p* < 0.05). **D** Dose-dependent fluorescent signal generated by tPA^488^ (green) and BSA^555^ (magenta) after SDS-PAGE. **E** Representative confocal photomicrographs of CP explants exposed 15 min to 40 nM of tPA^488^ (green), BSA^555^ (magenta) and DAPI staining (white). Scale bar 40 µm and 30 µm in the magnified inserts. **F** Graph shows quantification of fluorescence in CP explants (n = 5, mean ± SEM), * Significant difference (*p* < 0.05)

The potential transport of tPA through the CPs and its underlying mechanisms were investigated by tracking human recombinant tPA tagged with AlexaFluor^555^ (tPA^555^) or AlexaFluor^488^ (tPA^488^). We first used a model of primary culture of mouse CPECs (based on [30]. After 8 days, cultured CPECs display characteristics identical to those of the CPs in situ (Fig. 1A), namely a cuboid morphology, the expression of the marker transthyretin (TTR) and the presence of tight junctions (occludin staining). Cultured CPECs were able to uptake extracellular tPA^555^ (40 nM) in a time-dependent process (visible as increasing numbers of intracellular Alexa^555^ punctiform stainings, Fig. 1B). Measures of tPA^555^ levels in protein extracts from cell monolayers resolved by electrophoresis (Fig. 1C) confirmed these observations, with a sharp rise up to 5 min after initiating exposure, and an ongoing increase up to 60 min (after 5 min, the amount of internalized tPA^555^ already corresponded to 41.84% of what was internalized after 60 min). CPECs were also exposed for 15 min to doses of tPA^555^, increasing from 20 to 400 nM. Intracellular fluorescent dots accumulated when increasing doses of tPA^555^ (Fig. 1D). In-gel quantifications confirmed that the uptake of tPA^555^ in CPECs rose with the amount of tPA^555^ applied in the medium (after exposure to 20 nM, the amount of internalized tPA^555^ corresponded to 11.72% of what was internalized with 400 nM) (Fig. 1E). To support these findings, freshly isolated explants of mouse CPs were exposed to tPA^555^ and BSA^488^, used as a control (Bovine Serum Albumin is also a circulating protein, with a molecular weight comparable to that of tPA, as shown on Fig. 2A). CPECs exhibited strong accumulation of tPA^555^, but barely no signal for BSA^488^ (Fig. 2B, C). Inverting fluorophores on our tracked proteins gave similar results (Fig. 2D–F). In addition, we observed that 30 min after their intravenous co-injection (50 µg each), while tPA^555^ was detected in CPECs, Alexa^488^ extravasated out of choroidal vessels, but remained in the stroma without being taken up by CPECs (Additional file 1: Figure S1). Together, these in vitro and ex vivo results show that, CPECs uptake tPA, in a time- and dose-dependent manner, but do not uptake BSA.

**Fig. 3:**
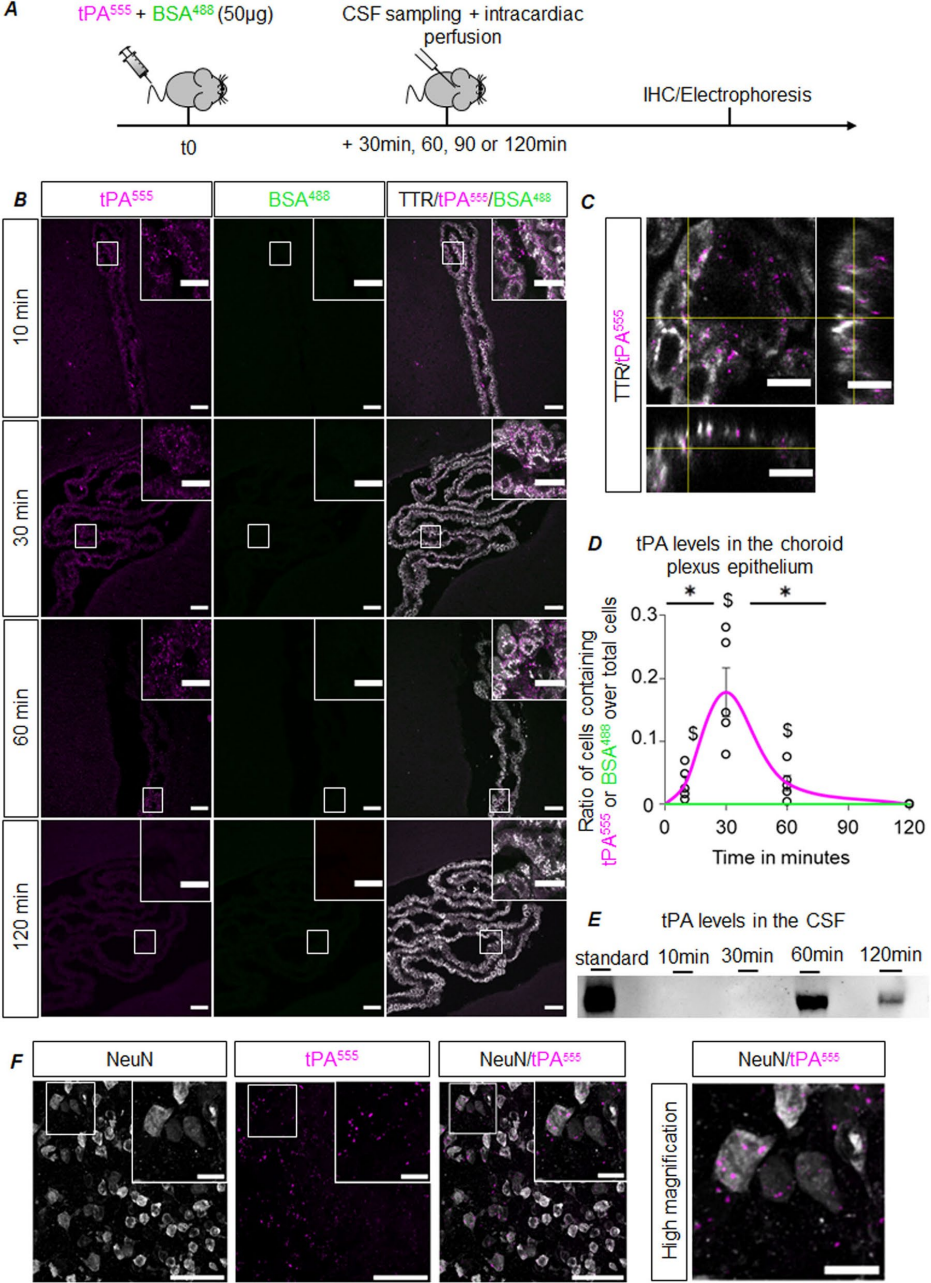
CPECs uptake tPA in vivo. **A** Experimental design: mice were injected intravenously with 50 µg of tPA^555^ and BSA^488^, CSF and brains were harvested 10, 30, 60 or 120 min later for protein analysis by electrophoresis and immunohistochemistry, respectively. **B** Confocal photomicrographs of CPs sections (representative of n = 5) with tPA^555^ (magenta), BSA^488^ (green) and TTR (white) staining, scale bar 40 µm and 20 µm in the magnified inserts. **C** Confocal photomicrograph of a CP section (representative of n = 5), tPA^555^ (magenta) and TTR (white) staining, panels show projection over the XZ axis (bottom) and over the YZ axis (right), scale bar 10 µm. **D** Graph shows mean ratio ± SEM (n = 5) of CPECs containing tPA^555^ or BSA^488^ over total CPECs. The magenta curve corresponds to tPA^555^ and the green one to BSA^488^, * significant difference (*p* < 0.05), $ significant difference from 0 and 120 min (*p* = 0.008). **E** Western-Blot for tPA levels in the CSF samples at each time-point. **F** Confocal photomicrographs acquired in the brain parenchyma (representative of n = 5) with tPA^555^ (magenta) and NeuN (white) staining, scale bar 40 µm and 10 µm in the magnified inserts

Then, mice were injected intravenously with 50 µg of tPA^555^ and 50 µg of BSA^488^, and CSF and brains were harvested 10, 30, 60 or 120 min later (Fig. 3A). In CPs, confocal microscopy (Fig. 3B) revealed dots of tPA^555^ as early as from 10 min, being more numerous at 30 and 60 min, and then virtually absent 120 min after intravenous injection. A z-stack of confocal images clearly shows that dots of tPA^555^ were located inside CPECs (Fig. 3C). We also found that tPA was internalized in clathrin-coated endocytotic vesicles, as it was co-localized with the marker AP2 (Pearson’s coefficient of 65%; Additional file 1: Figure S2). We did not find any BSA^488^ in CPs after intravenous injection. We quantified the number of TTR immune-positive cells with dots of tPA^555^ as an index of the internalization of tPA by CPECs (Fig. 3D)*.* This index followed a bell curve shape, peaking 30 min post-injection. After two hours, tPA was no longer detected in the CPs, suggesting a passage of tPA^555^ from the blood to the CSF. Accordingly, an immunoblot analysis on CSF samples harvested at different time points, revealed the appearance of tPA in the CSF starting between 30 and 60 min post intravenous injection (Fig. 3E). Since the antibody used is specific for human tPA and does not recognize murine tPA, this confirms that vascular tPA crosses the CPs and reaches the CSF. Of note, when administered in the ventricle, tPA^555^ was also detected in the CPECs, suggesting that CPs also allow the clearance of tPA from the CSF (Additional file 1: Figure S3). We also found that after an intravenous injection, the decline in tPA^555^ levels in the blood parallels the increase in tPA^555^ levels in the CSF (Additional file 1: Figure S4). Moreover, to ensure that the observed transport occurs directly from the choroidal circulation to the CPECs, and not via indirect pathways, we administered tPA^555^ in the internal carotid and demonstrated that very shortly after (5 min), dots of tPA^555^ were detected in the stroma and in CPECs (Additional file 1: Figure S5). Importantly, in barrier-protected parenchymal areas, we could detect tPA^555^ in neurons, suggesting that once in the CSF, tPA can enter the parenchyma (Fig. 3F). In barrier-free areas, both tPA^555^ and BSA^488^ reached the brain parenchyma (exemplified here in the median eminence: 30 min after injection, fluorescent BSA and tPA diffused outside the vascular system, Additional file 1: Figure S6).

Overall, these results show that after an intravenous injection, tPA is internalized by CPECs and is then transported to the CSF. This suggests that CPs are a potential route of entry for tPA into the brain parenchyma.

### The uptake of tPA by choroid plexus epithelial cells is mediated by LRP1

**Fig. 4:**
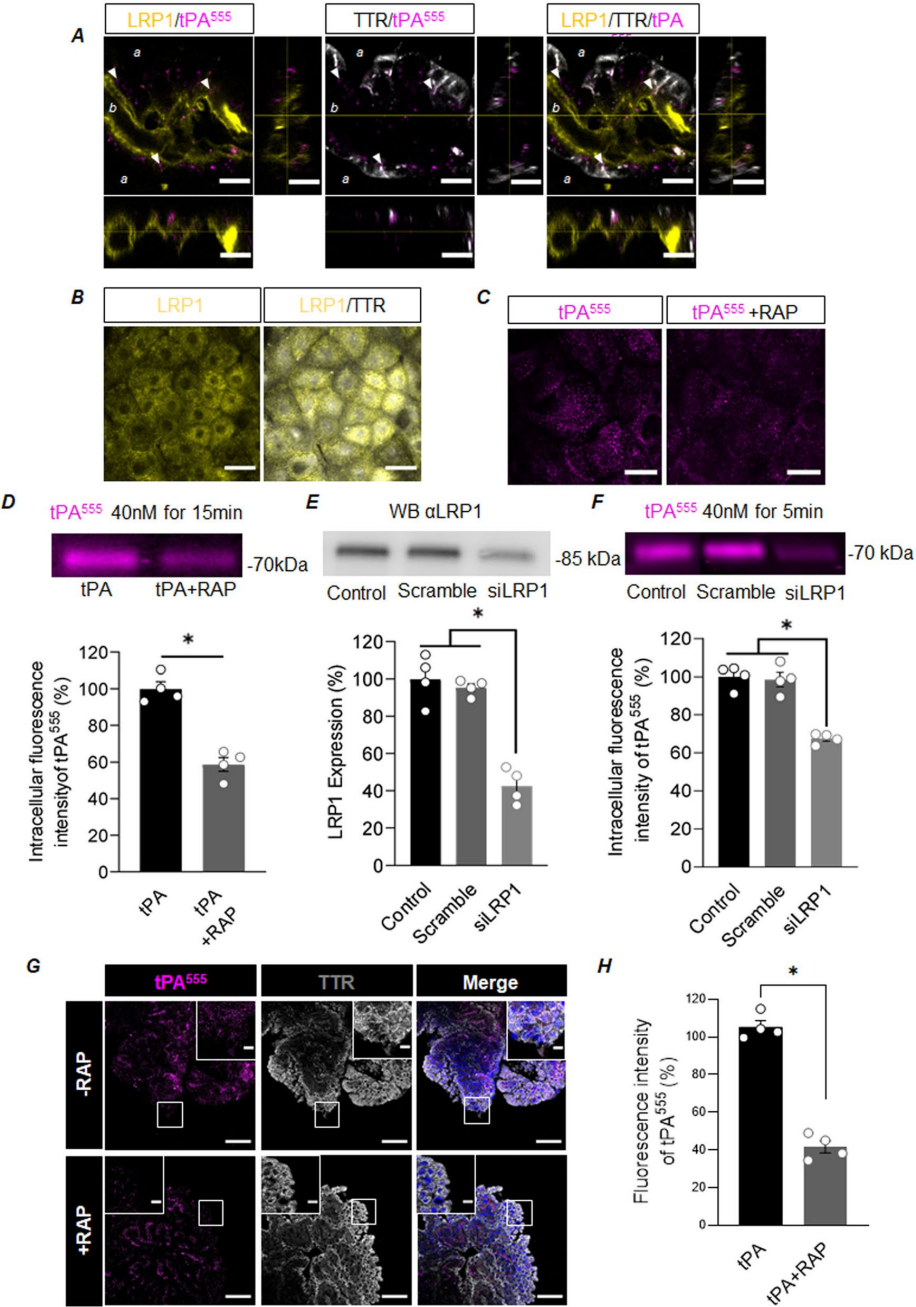
Internalization of tPA CPECs is an active process, mediated by LRP1. **A** Representative photomicrographs (n = 5) of CPs harvested from mice intravenously injected with tPA^555^ (tPA^555^ in magenta, LRP1 in yellow, and TTR in white); panels show projection over the XZ axis (bottom) and over the YZ axis (right), scale bar 10 µm. Arrowheads point examples of tPA^555^ dots that can be found close to LRP1 staining in TTR positive cells. a: apical side; b: basal side. **B** Representative photomicrographs (n = 4) of cultured CPECs show LRP1 (yellow) and TTR (white), scale bar 40 µm. **C** Representative photomicrographs (n = 4) of CPECs exposed for 15 min to tPA^555^ (40 nM) alone or with RAP (500 nM), scale bar 40 µm. **D** Representative electrophoresis (representative of n = 4) and corresponding quantification (mean ± SEM, * significant difference (*p* < 0.05)), of protein extracts from monolayer of CPECs exposed for 15 min to tPA^555^ (40 nM) alone or with RAP (500 nM). **E** Representative western blot against LRP1 and corresponding quantification after transfection of cultured CPECs with nothing (Control), negative control (Scramble) or siRNA anti-LRP1 (siLRP1) (n = 4, mean ± SEM of LRP1 expressed as percentage of control). **F** Representative electrophoresis and corresponding quantification of protein extracts from monolayer of CPECs exposed for 15 min to tPA^555^ (40 nM) after transfection with control, scramble or siLRP1 (n = 4, mean ± SEM of LRP1 expressed as percentage of control). **G**–**H** mice were injected intravenously with 50 µg of tPA^555^ alone or together with 90 µg of RAP. 30 min later, PCs were freshly isolated for immunohistochemistry. **G** Representative confocal photomicrographs (n = 4) of a stack of CPs explants, with tPA^555^ (magenta), TTR (white) and DAPI (blue) staining. **H** Quantifications of tPA^555^ fluorescence intensity in CPs explants. Scale bar 50 µm and 10 µm in the magnified inserts

Transcytosis/uptake of tPA has been attributed in various cell types to members of the LRP family, in particular to LRP1 [3, 4, 14]. A 3D projection of confocal images acquired after intravenous injection of tPA^555^ in mice and coupled to the immunodetection of LRP1 on TTR positive cells supports the hypothesis that LRP1 could be responsible for tPA transport by CPECs. Indeed, we found dots of tPA^555^ close to LRP1 immunolabellings at the basolateral pole of CPECs (Fig. 4A). In cultured CPECs, that also express LRP1 (Fig. 4B), we first found that tPA^555^ internalization was likely an active mechanism. Indeed, when exposed to 40 nM of tPA^555^ for 15 min, CPECs protein extracts displayed fluorescent signals when exposure was performed at 37 °C but not when performed at 4 °C (data not shown). To evaluate the participation of a member of the LRP family, we compared the internalization of tPA by CPECs in the absence or presence of RAP (Receptor Associated Protein, 500 nM), a competitive LRP antagonist (Fig. 4C–D). CPECs co-exposed to tPA^555^ and RAP presented fewer tPA^555^ dots than cells exposed to tPA^555^ alone (Fig. 4C). This result was confirmed by electrophoresis, showing a 41.22% reduction in tPA^555^ internalization induced by RAP co-treatment (Fig. 4D). To evaluate the participation of LRP1 in the uptake of tPA by the CPECs, we knocked-down its expression with a siRNA strategy. The extinction of LRP1 expression was 58.77% effective (Fig. 4E). When compared to control CPECs or CPECs transfected with a scramble siRNA, CPECs transfected with LRP1 siRNA internalized 32.55% less tPA^555^ (Fig. 4F). Importantly, in vivo, we confirmed the role of LRP1 in transporting tPA in CPECs. Indeed, there was around 60% loss of tPA^555^-related fluorescence in CPECs when RAP (90 µg) was co-administered intravenously with tPA^555^ (50 µg), compared to tPA^555^ alone (Fig. 4G–H).

### The finger domain of tPA is critical for the passage of tPA across choroid plexus epithelial cells

**Fig. 5:**
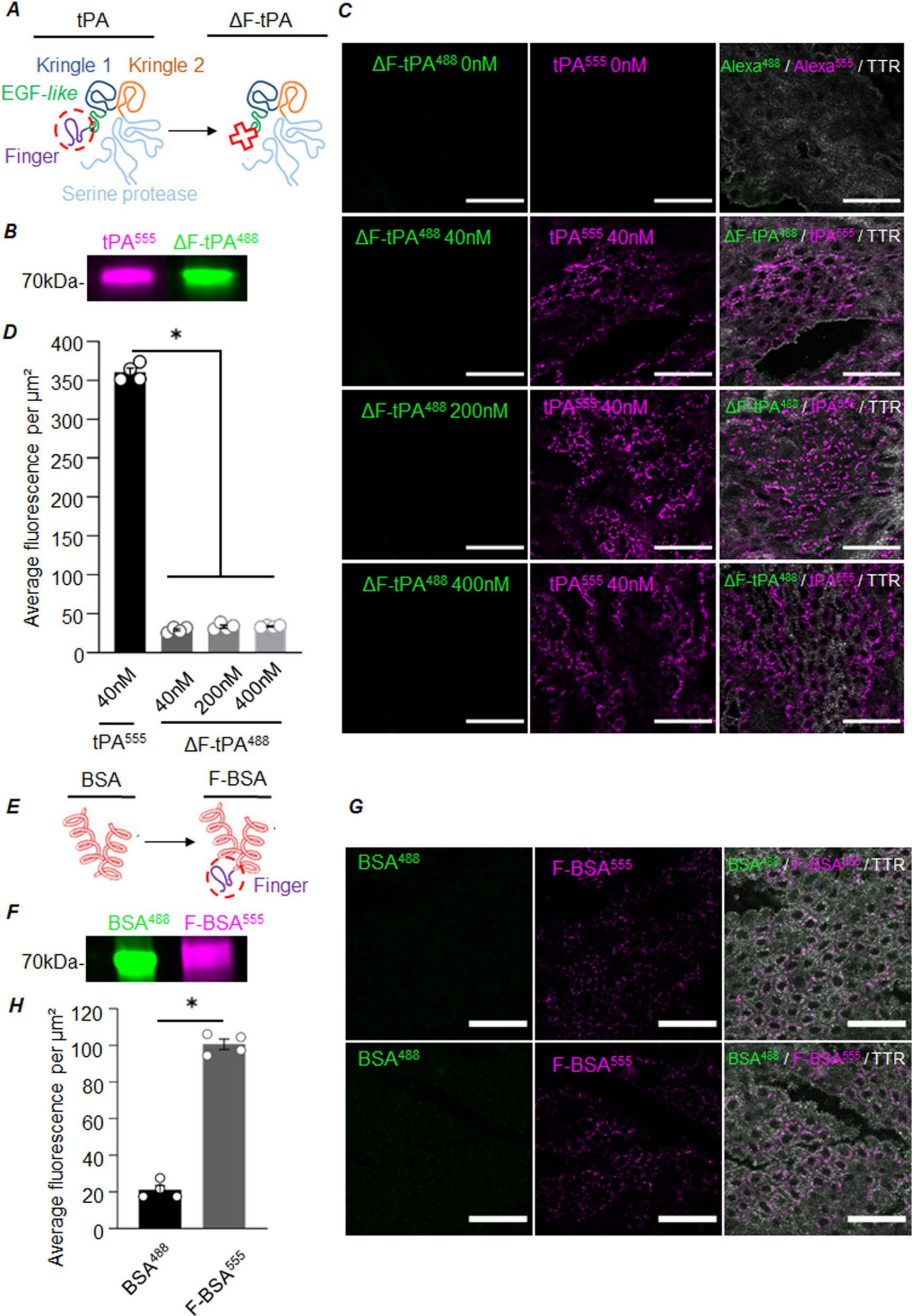
The Finger domain of tPA allows the internalization of tPA and BSA by CPECs ex vivo. **A** Schematic representation of the functional domains of tPA and its mutant form ΔF-tPA, deleted of the finger domain. **B** Electrophoresis of 5 µg of tPA^555^ (magenta) and ΔF tPA^488^ (green) in SDS-PAGE and revelation in fluorescence. **C**–**D** Representative photomicrographs and corresponding quantifications of CPs explants exposed to tPA^555^ (40 nM) and ΔF-tPA^488^ (40 nM, 200 nM or 400 nM) for 15 min (n = 4, mean ± SEM). **E** Schematic representation of BSA and its mutant form F-BSA, adding the finger domain of tPA. **F** Electrophoresis of 5 µg of BSA^488^ (green) and F-BSA^555^ (magenta) in SDS-PAGE and revelation in fluorescence. **G**, **H** Representative photomicrographs and corresponding quantifications of CPs explants exposed to BSA^488^ (40 nM) and F-BSA^555^ (40 nM) for 15 min (n = 4, mean ± SEM). TTR staining (white) is shown on the merged images (**C** and **F**). Scale bar: 40 µm

Among its five functional domains (Fig. 5A), the finger domain of tPA is known to be the one interacting with LRP1. To assess its involvement in the passage of CPECs, we produced a tPA devoid of its finger domain (ΔF-tPA), and alternatively, we grafted the finger domain of tPA to albumin (F-BSA) (Fig. 5E). Both mutant proteins were also tagged with an Alexa Fluor for their tracking (Fig. 5B, F). *Ex-vivo*, CP explants were exposed to 40 nM of tPA^555^ and with increasing doses of ΔF-tPA^488^ (40 nM, 200 nM or 400 nM) for 15 min (Fig. 5C, D). As expected, tPA^555^ was internalized by CPECs whereas ΔF-tPA was not, even with a tenfold higher concentration**.** The finger domain of tPA is therefore necessary for internalization by CPECs. Interestingly, fusing the finger domain of tPA to BSA conferred the new ability to BSA to be internalized by CPECs ex vivo (Fig. 5G, H).

**Fig. 6:**
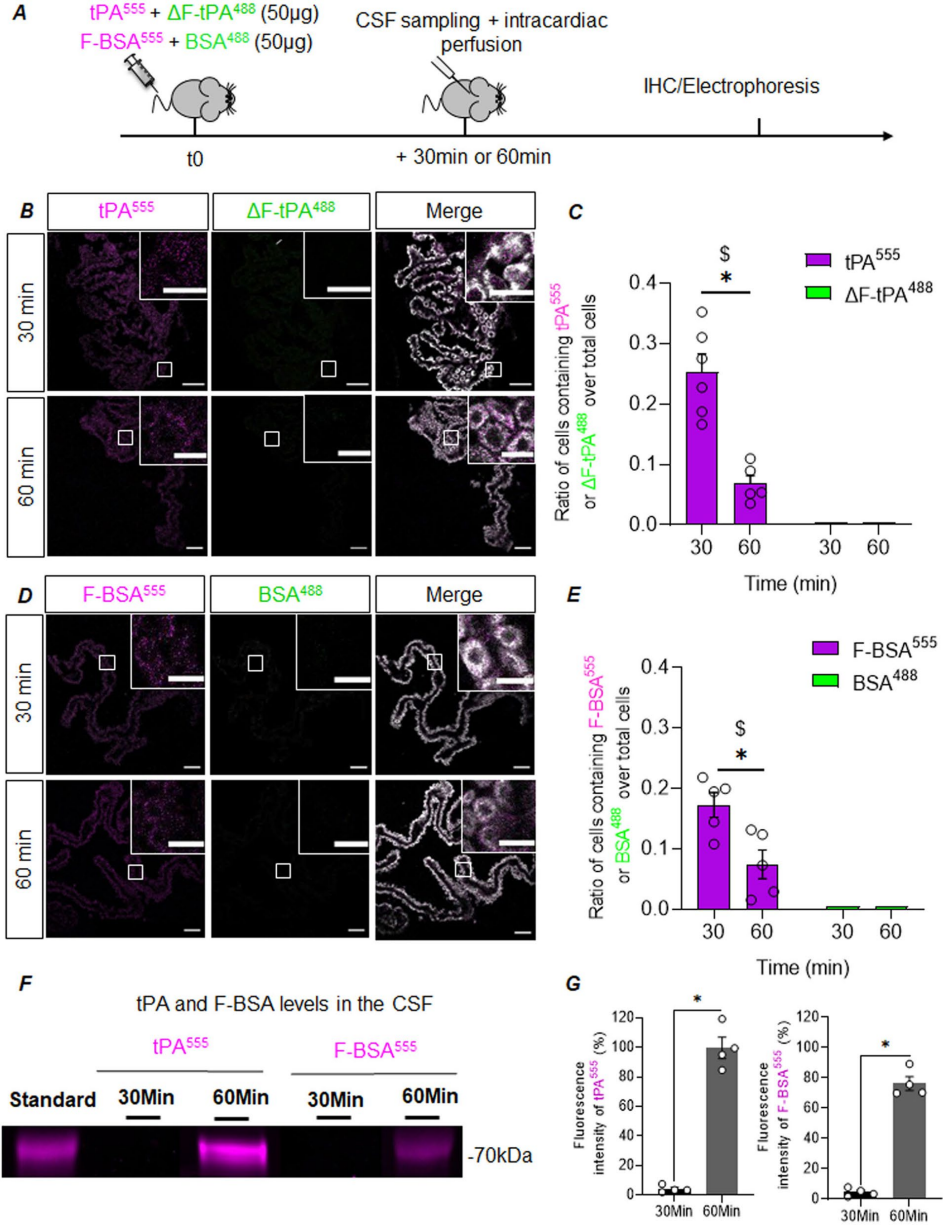
The Finger domain of tPA allows the transport of tPA and BSA across the BCSF barrier in vivo. **A** Experimental design: mice were injected intravenously with 50 µg of tPA^555/^ ΔF-tPA^488^ or F-BSA^555^/BSA^488^, CSF and brains were harvested 30 or 60 min later for protein analysis by electrophoresis and immunohistochemistry, respectively. **B**–**C**, Representative confocal photomicrographs and corresponding quantification (mean ratio of CPECs containing tPA^555^ or ΔF-tPA^488^ over total CPECs ± SEM, n = 5) of CPs sections with tPA^555^ (magenta), ΔF-tPA^488^ (green) and TTR (white) staining; scale bar 40 µm and 20 µm in the magnified inserts. * significant difference (*p* < 0.05), $ significant difference from 0 min (*p* = 0.0022). **D**–**E** Representative confocal photomicrographs and corresponding quantification (mean ratio of CPECs containing tPA^555^ or ΔF-tPA^488^ over total CPECs ± SEM, n = 5) of CPs sections with F-BSA^555^ (magenta), BSA^488^ (green) and TTR (white) staining; scale bar 40 µm and 20 µm in the magnified inserts. * significant difference (*p* < 0.05), $ significant difference from 0 min (*p* = 0.0079). **F**–**G**, Representative electrophoresis and corresponding quantification for tPA^555^ and F-BSA^555^ levels in CSF samples at 30 and 60 min (mean ± SEM, n = 4)

These ex vivo observations were confirmed in vivo, in mice injected intravenously with 50 µg of tPA^555^/ ΔF-tPA^488^ or with 50 µg of F-BSA^555^/ BSA^488^ (Fig. 6A). A confocal microscopy analysis (Fig. 6B) revealed dots of tPA^555^ in TTR positive cells, 30 and 60 min after injection. However, virtually no dots were detected for ΔF-tPA^488^. We quantified the number of TTR immune-positive cells with dots of tPA^555^ and dots of ΔF-tPA^488^ as an index of their internalization by CPECs (Fig. 6C)*.* This index was null for ΔF-tPA^488^ and peaked 30 min post-injection of tPA^555^, with 73.09% more cells containing tPA^555^ than at 60 min. Interestingly, the confocal microscopy analysis (Fig. 6D) revealed dots of F-BSA^555^ in TTR positive cells, 30 and 60 min after IV injection, while no dots were detected for BSA^488^. The corresponding quantification confirmed that BSA per se, cannot be uptaken by CPECs, but that grafting the finger domain of tPA then allows the internalization of the fusion protein (Fig. 6E)*.* This index followed a bell curve shape, with a peak of internalization of tPA^555^ reached 30 min post-injection, with 56.84% more cells containing tPA^555^, than at 60 min and no detection of ΔF-tPA^488^. Also important, as confirmed by electrophoresis of corresponding CSF samples, F-BS^A555^ can cross the BCSFB, like tPA^555^ (Fig. 6F, G). The finger domain of tPA is thus an interesting strategy to allow therapeutic fusion proteins reaching the CSF and the brain parenchyma.

## Discussion

Besides ensuring the adequate cerebral perfusion, circulating tPA controls brain physiopathology, at least by a direct action on the neurovascular unit. Here, we show that in addition to the BBB [3], the BCSFB is also a door to the brain for circulating tPA. Thus, together with endothelial and parenchymal cells that produce tPA, both the BBB and the BCSFB contribute to modulate the levels of tPA in the different compartments of the brain.

When considering inward flux to the brain, CPs have two interesting features: the presence of fenestrations between choroidal endothelial cells and a rich vascular density. Vascular peptides/proteins (even of several hundred kDa) can cross the highly permeable choroidal endothelium via either fenestrae or vesicular shuttling. When reaching the CPEC basement membrane, some peptides/proteins might enter the CSF via paracellular convection and diffusion through apical tight junctions, while other could undergo transcytosis by pinocytosis, or via a specific receptor-mediated process, including the transferrin receptor, the insulin receptor or members of the low-density lipoprotein (LDL) receptor family. Here, we identified LRP1 of CPEC as a mediator of the transport of tPA from the blood to the CSF.

The lack of polarity of cultured CPECs and the fact that tPA exposure on explants occurs at both sides, do not allow distinguishing influx and efflux processes. In vivo, we demonstrate that both influx and efflux of tPA through the CPs exist. Aβ, has also been shown to be transported and/or taken up by CPs when present either at the apical or basal side, with a preferred CSF-to-blood direction [16]. Thus a single molecule can be internalized and/or transported from both CSF and blood, not only depending on its gradient of concentration, but also on energy dependent systems. The relative efficacy of influx versus efflux transport, which may vary depending on tPA levels circulating in blood and CSF would deserve additional investigation. The relative delay of detection of tPA in the CSF after intravenous administration may suggest that it could come from alternative routes. But since tPA can be found in the stroma and in CPECs very early after delivery through the carotid artery, the CP is undoubtedly an interface allowing the transport of tPA from the blood to the CSF.

Several members of the LDL receptor family have been identified in the CPs, including the LDL receptor (LDLR), LRP1, LRP2 (megalin/gp 330, although probably at low levels [8], and LRP8/ApoE receptor 2 [41]. None of these has been investigated in relationship with tPA transport in the CPs. Instead, they have received more attention in the context of Alzheimer’s disease, in particular LRP1 and LRP2 of which Aβ, like tPA, is a ligand. In this context, age and/or disease-related alterations in LRP1 and LRP2 levels could reduce the efflux of Aβ and concourse to the pathogenesis of Alzheimer’s disease [11, 27, 40]. Together with PgP, LRP1 is believed to drive the efflux of Aβ from the CSF to the blood while LRP2 would drive the influx of Aβ, together with RAGE. This would imply that LRP1 is expressed at the apical side and LRP2 at the basolateral side of CPECs, which would not be compatible with our findings of an influx of tPA mediated by LRP1. In fact, the literature about the subcellular localization of LRP on CPECs is inconsistent [7, 11, 39, 47]. In our hands, in vivo immunostainings for LRP1 were stronger at the basal/basolateral sides. Thus, our findings support the hypothesis of a LRP1-dependent influx of tPA, while the efflux may be LRP-1 independent. Altogether, without excluding other mechanisms, our data show that the LRP-1 driven uptake is an important pathway of transport of vascular tPA in CPECs.

Together, the BBB and BCSFB prevent the parenchymal passage of all but a fraction of small uncharged molecules. Accordingly, parenteral delivery results in low levels of drugs in the CNS, which likely limits their efficacy. Interestingly, low energy shockwave pulse has recently been shown to increase the permeation of the BCSFB to some molecules [24]. Alternative routes of administration including direct delivery into the CSF (intrathecal administration) or into the parenchyma (convection-enhanced delivery) might overcome this limit of parenteral delivery, but are invasive and risky (for review see [15]. Receptor-mediated transcytosis may be another promising mechanism to deliver protein through CNS barriers. For instance, molecular trojan based on the fusion of therapeutic protein motifs to antibodies against the transferrin receptor or the insulin receptor allow brain delivery of otherwise non-transportable proteins [23]. The present study adds to these approaches and opens interesting prospects. We show that vascular tPA can cross the BCSFB via LRP1-mediated transcytosis, as demonstrated earlier at the BBB level [3] and [4]. Together, both the BBB and BCSFB pathways may allow tPA accessing neurons to modulate their activity. We also determine that among its five functional domains, the finger domain of tPA is mandatory for the transport of tPA across the CPs. Depending on the context, it could be interesting to open, or conversely, to close these two roads to the brain for circulating tPA. For instance, in vasculo-occlusive conditions, engineering a tPA deleted of its finger domain could increase its availability in the blood, together with limited side-effects on the neurovascular unit. On the other hand, some conditions likely require the presence of tPA in the CSF. For instance, reduced tPA CSF levels in AD patients (versus patients with subjective or mild cognitive impairment) have been suggested to contribute to some neurodegenerative pathways of the disease [19]. Restoring tPA levels in the CSF via vascular delivery might counteract this.

Another interesting finding is that while albumin is not normally transported through the CPECs, fusion to the finger domain of tPA this time allowed passage from the blood to the CSF via CPECs. Further studies should explore the actual therapeutic efficacy of such a strategy on protein molecules with known protective actions on brain cells, but unable to reach their target after iv injection.

## Conclusions

To summarize, our study shows that the choroid plexus is a door to the brain for vascular tPA. By dissecting the molecular determinants of the passage of tPA across the choroidal epithelium, we uncover opportunities to modulate exchanges from the blood to the central nervous system.

## Supplementary Information

**Additional file 1: Figure S1. **In contrast to tPA coupled to Alexa, Alexa alone is not taken-up by CPECs *in vivo*.** A, **Experimental design: mice were injected intravenously with of tPA^555^ and Alexa^488^ (50µg each), brains were harvested 30min later for immunohistochemistry.** B, **Representative confocal photomicrographs (n=4) of CPs sections with tPA^555^ (magenta), Alexa^488^ (green) and DAPI (blue) staining; scale bar 50µm (upper panels) and 10µm in the magnified inserts (lower panels). **Figure S2. **CPECs internalized tPA by clathrin-coated endocytosis vesicles.* A*, Confocal photomicrographs (representative of n=6 slices) of CPs sections with tPA^555^ (left bottom, magenta), TTR (left top, white) and AP2 (right top, green) staining; scale bar 50µm and 10µm in the magnified inserts.*** B***, Image analysis of confocal photomicrographs using the Imaris Microscopy Image Analysis Software to study the colocalization between tPA^555^ and AP2 staining (yellow, right). tPA endocytosis (white arrow); scale bar 10µm. **Figure S3.** Existence of an efflux pathway for tPA. **A**, Experimental design: mice received an intra-cerebroventricular injection of tPA^555^ (1µl at 0.9mg/ml), and brains were harvested 10, 30 or 60min later for immunohistochemistry. **B**, Representative confocal photomicrographs (n=4) of CPs sections with tPA^555^ (magenta), LRP1 (green), TTR (white) and DAPI (blue) staining; scale bar 50µm and 10µm in the magnified inserts. **Figure S4.** Evolution of tPA^555^ levels in the blood and CSF. **A**, Experimental design illustrating that mice were injected intravenously with 50µg of tPA^555^ and BSA^488^, CSF, blood and brains were harvested 15, 30, or 45min later for protein analysis by electrophoresis (2µl of CSF and 1 µl of plasma were resolved for each animal). **B**, Representative electrophoreses and **C**, corresponding quantification for tPA^555^ levels in the plasma and in the CSF (mean ±SEM, n=5). ** significant difference (p<0.005). **Figure 5: **tPA^555^ is rapidly taken-up by CPECs after intra-carotid injection. **A**, Experimental design: mice were injected in the carotid with 50µg of tPA^555^, brains were harvested 5min later, and PCs freshly isolated for immunohistochemistry. **B**,** C**,** D**: Representative confocal photomicrographs (n=5) of a stack of CPs explants with tPA^555^ (magenta) and DAPI staining (blue), focus on PCs vasculature (**B**), on PCs stroma (**C**) and on CPECs (**D**). Scale bar 50µm and 10µm in the magnified inserts. **Figure S6. **Confocal photomicrograph shows tPA^555^ (magenta) and BSA^488^ (green) extravasation out of vessels (CD31 staining in white), in the Median Eminence, a barrier free zone; DAPI staining (blue), scale bar 40µm

## Data Availability

The datasets analyzed during the current study are available from the corresponding author on request.

## Abbreviations

BBB: Blood–brain-barrier
CSF: Cerebrospinal fluid
BCSFB: Blood-CSF-barrier
CPs: Choroid plexuses
CPECs: Choroid plexus epithelial cells
LRP: Low density lipoprotein receptor-related protein
TPA: Tissue-type plasminogen activator
TTR: Transthyretin
