# Correction to: The choroid plexus: A door between the blood and the brain for tissue-type plasminogen activator

**DOI:** 10.1186/s12987-022-00383-3

**Published:** 2022-11-01

**Authors:** Vincent Zuba, Jonathane Furon, Mathys Bellemain-Sagnard, Sara Martinez de Lazarrondo, Laurent Lebouvier, Marina Rubio, Yannick Hommet, Maxime Gauberti, Denis Vivien, Carine Ali

**Affiliations:** 1 Physiopathology and Imaging of Neurological Disorders, Normandie Univ, UNICAEN, INSERM, INSERM, UMR-S U1237, Institut Blood and Brain @ CaenNormandie, GIP Cyceron, Boulevard Becquerel, 14074 Caen, France; 2 Department of Clinical Research, Caen-Normandie Hospital (CHU), Caen, France

**Correction to: Fluids Barriers CNS 19, 80 (2022).**

10.1186/s12987-022-00378-0

The original publication of this article contained an incorrect author name. The incorrect and correct name are shown below. The original article has been updated.

**Author name**.

- The incorrect author name is: Sara Martinez de Lazarrondo.
- The correct author name is: Sara Martinez de Lizarrondo.

