# Supplementary material for: The choroid plexus: a door between the blood and the brain for tissue-type plasminogen activator
Source: Fluids Barriers CNS. 2022 Oct 15;19:80. doi: 10.1186/s12987-022-00378-0 (PMC9569045; doi:10.1186/s12987-022-00378-0)

**Supplementary materials**

**Material and methods:**

***Co-localization study***

To determine if tPA was internalized via clathrin-coated endocytosis vesicles, CPs harvested 10 min after injection of tPA^555^, fixed and immunostained for the AP2 adaptator complex (1:500, Abcam Ab2730). Images acquired by confocal microscopy were also analysed using the *Imaris* Microscopy Image Analysis Software, to ensure co-localization between tPA and AP2 via the calculation of the Pearson’s coefficient (from 6 different slices/ROIs).

***Intracerebroventricular injection of Alexa-coupled recombinant tPA***

Anesthetized mice were placed in a stereotaxic frame. Lidocaine (a local pain reliever) was applied on the incision plan 5 minutes before surgery. The skin was incised in order to unmask the skull a small craniotomy was performed (coordinates -0.2mm anteroposterior; +1mm lateral; -2mm depth from bregma). A glass micropipette containing 1µL of Alexa-coupled recombinant tPA (0.9 mg/mL) was inserted and the content was gently injected in the left lateral ventricle at a rate of 0.25µL/min. The glass pipette was left in place 2 min after injection and slowly removed to prevent reflux.

***Intra-carotidian injection of Alexa-coupled recombinant tPA***

Anesthetized mice were placed on the back and a midline incision was performed in the neck. The Right Common Carotid Artery was isolated. A Piece of Whatman ™ Filter Paper Strip was placed under the artery, then a catheter was inserted. 50µg of tPA^555^ were injected in 200µL of saline (0.9% NaCl) over approximately 5 seconds. Afterwards, brains were harvested, and PCs freshly isolated. PCs were rinsed three times with PBS, fixed with 4% paraformaldehyde in PBS 0.1 M and pH 7.4, for 15min, placed on poly-lysine slides, mounted in Fluoromount-G®.

**Legends**

**Additional file Figure 1:** In contrast to tPA coupled to Alexa, Alexa alone is not taken-up by CPECs *in vivo*. **A,** Experimental design: mice were injected intravenously with of tPA^555^ and Alexa^488^ (50µg each), brains were harvested 30min later for immunohistochemistry. **B,** Representative confocal photomicrographs (n=4) of CPs sections with tPA^555^ (magenta), Alexa^488^ (green) and DAPI (blue) staining; scale bar 50µm (upper panels) and 10µm in the magnified inserts (lower panels).


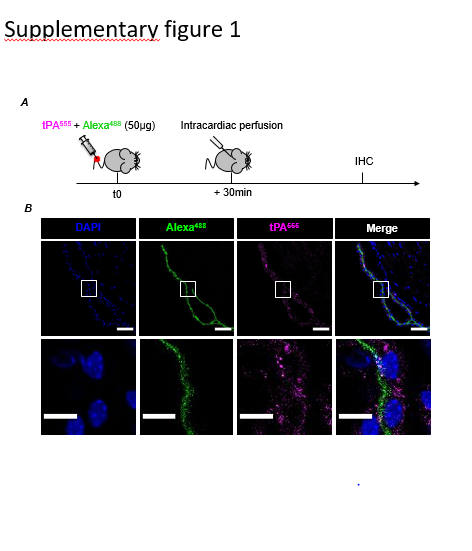


**Additional file Figure 2:** CPECs internalized tPA by clathrin-coated endocytosis vesicles. **A**, Confocal photomicrographs (representative of n=6 slices) of CPs sections with tPA^555^ (left bottom, magenta), TTR (left top, white) and AP2 (right top, green) staining; scale bar 50µm and 10µm in the magnified inserts. **B**, Image analysis of confocal photomicrographs using the Imaris Microscopy Image Analysis Software to study the colocalization between tPA^555^ and AP2 staining (yellow, right). tPA endocytosis (white arrow); scale bar 10µm.


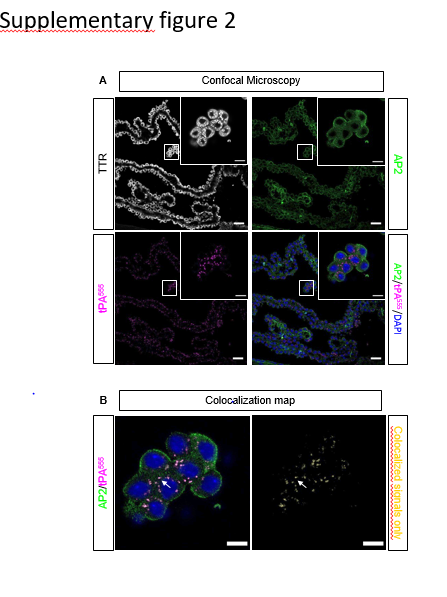


**Additional file Figure 3:** Existence of an efflux pathway for tPA. **A**, Experimental design: mice received an intra-cerebroventricular injection of tPA^555^ (1µl at 0.9mg/ml), and brains were harvested 10, 30 or 60min later for immunohistochemistry. **B**, Representative confocal photomicrographs (n=4) of CPs sections with tPA^555^ (magenta), LRP1 (green), TTR (white) and DAPI (blue) staining; scale bar 50µm and 10µm in the magnified inserts.


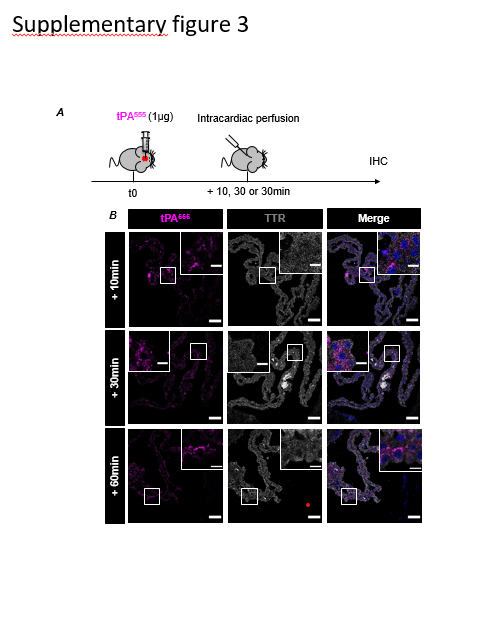


**Additional file Figure 4:** Evolution of tPA^555^ levels in the blood and CSF. **A**, Experimental design illustrating that mice were injected intravenously with 50µg of tPA^555^ and BSA^488^, CSF, blood and brains were harvested 15, 30, or 45min later for protein analysis by electrophoresis (2µl of CSF and 1 µl of plasma were resolved for each animal). **B**, Representative electrophoreses and **C**, corresponding quantification for tPA^555^ levels in the plasma and in the CSF (mean ±SEM, n=5). ** significant difference (p<0.005)

**
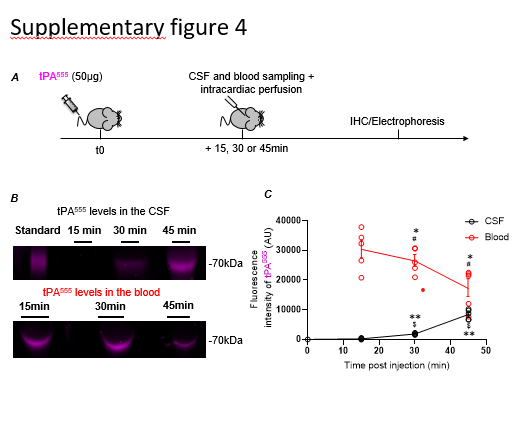
**

**Additional file Figure 5:** tPA^555^ is rapidly taken-up by CPECs after intra-carotid injection.

**A**, Experimental design: mice were injected in the carotid with 50µg of tPA^555^, brains were harvested 5min later, and PCs freshly isolated for immunohistochemistry. **B**, **C**, **D**: Representative confocal photomicrographs (n=5) of a stack of CPs explants with tPA^555^ (magenta) and DAPI staining (blue), focus on PCs vasculature (**B**), on PCs stroma (**C**) and on CPECs (**D**). Scale bar 50µm and 10µm in the magnified inserts.


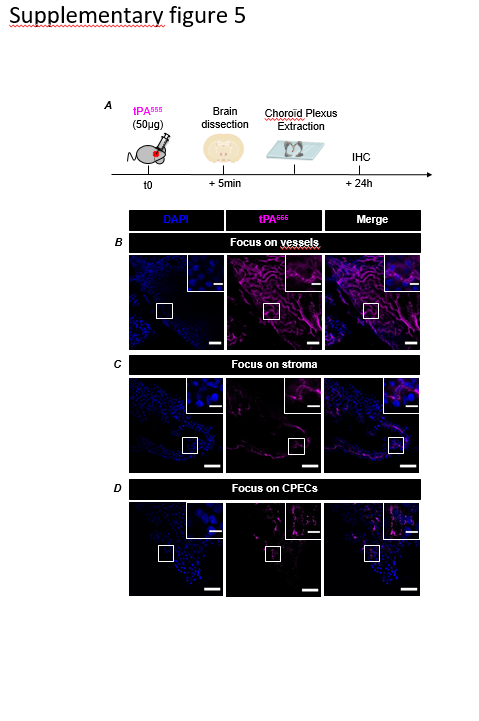


**Additional file Figure 6:** Confocal photomicrograph shows tPA^555^ (magenta) and BSA^488^ (green) extravasation out of vessels (CD31 staining in white), in the Median Eminence, a barrier free zone; DAPI staining (blue), scale bar 40µm.


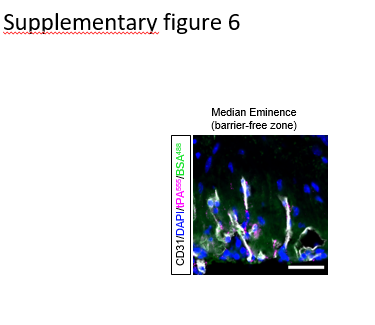

Supplement: Supplementary file 1 — Additional file 1: Figure S1. In contrast to tPA coupled to Alexa, Alexa alone is not taken-up by CPECs in vivo. A, Experimental design: mice were injected intravenously with of tPA555 and Alexa488 (50µg each), brains were harvested 30min later for immunohistochemistry. B, Representative confocal photomicrographs (n=4) of CPs sections with tPA555 (magenta), Alexa488 (green) and DAPI (blue) staining; scale bar 50µm (upper panels) and 10µm in the magnified inserts (lower panels). Figure S2. CPECs internalized tPA by clathrin-coated endocytosis vesicles. A, Confocal photomicrographs (representative of n=6 slices) of CPs sections with tPA555 (left bottom, magenta), TTR (left top, white) and AP2 (right top, green) staining; scale bar 50µm and 10µm in the magnified inserts. B, Image analysis of confocal photomicrographs using the Imaris Microscopy Image Analysis Software to study the colocalization between tPA555 and AP2 staining (yellow, right). tPA endocytosis (white arrow); scale bar 10µm. Figure S3. Existence of an efflux pathway for tPA. A, Experimental design: mice received an intra-cerebroventricular injection of tPA555 (1µl at 0.9mg/ml), and brains were harvested 10, 30 or 60min later for immunohistochemistry. B, Representative confocal photomicrographs (n=4) of CPs sections with tPA555 (magenta), LRP1 (green), TTR (white) and DAPI (blue) staining; scale bar 50µm and 10µm in the magnified inserts. Figure S4. Evolution of tPA555 levels in the blood and CSF. A, Experimental design illustrating that mice were injected intravenously with 50µg of tPA555 and BSA488, CSF, blood and brains were harvested 15, 30, or 45min later for protein analysis by electrophoresis (2µl of CSF and 1 µl of plasma were resolved for each animal). B, Representative electrophoreses and C, corresponding quantification for tPA555 levels in the plasma and in the CSF (mean ±SEM, n=5). ** significant difference (p<0.005). Figure 5: tPA555 is rapidly taken-up by CPECs after intra-carotid injection. [file 12987_2022_378_MOESM1_ESM.docx]
